# Herpes zoster sciatica mimicking lumbar canal stenosis: a case report

**DOI:** 10.1186/s13104-015-1272-z

**Published:** 2015-07-29

**Authors:** Masao Koda, Chikato Mannoji, Makiko Oikawa, Masazumi Murakami, Yuzuru Okamoto, Tamiyo Kon, Akihiko Okawa, Osamu Ikeda, Masashi Yamazaki, Takeo Furuya

**Affiliations:** Department of Orthopedic Surgery, Graduate School of Medicine, Chiba University, 1-8-1 Inohana, Chuo-Ku, Chiba, 260-8670 Japan; Department of Orthopedic Surgery, Chiba Aoba Municipal Hospital, Chiba, Japan; Department of Dermatology, Chiba Aoba Municipal Hospital, Chiba, Japan; Department of Orthopedic Surgery, Tsukuba University, Ibaraki, Japan

**Keywords:** Herpes zoster, Lumbar spinal canal stenosis, Diagnosis

## Abstract

**Background:**

Symptom of herpes zoster is sometimes difficult to distinguish from sciatica induced by spinal diseases, including lumbar disc herniation and spinal canal stenosis. Here we report a case of sciatica mimicking lumbar canal stenosis.

**Case presentation:**

A 74-year-old Chinese male patient visited our hospital for left-sided sciatic pain upon standing or walking for 5 min of approximately 1 month’s duration. At the first visit to our hospital, there were no skin lesions. A magnetic resonance imaging showed spinal canal stenosis between the 4th and 5th lumbar spine. Thus, we diagnosed the patient with sciatica induced by spinal canal stenosis. We considered decompression surgery for the stenosis of 4th and 5th lumbar spine because conservative therapy failed to relieve the patient’s symptom. At that time, the patient complained of a skin rash involving his left foot for several days. A vesicular rash and erythema were observed on the dorsal and plantar surfaces of the great toe and lateral malleolus. The patient was diagnosed with herpes zoster in the left 5th lumbar spinal nerve area based on clinical findings, including the characteristics of the pain and vesicular rash and erythema in the 5th lumbar spinal dermatome. The patient was treated with famciclovir (1,500 mg/day) and non-steroidal anti-inflammatory drugs. After 1 week of medication, the skin rash resolved and pain relief was obtained.

**Conclusion:**

In conclusion, spinal surgeons should keep in mind herpes zoster infection as one of the possible differential diagnoses of sciatica, even if there is no typical skin rash.

**Electronic supplementary material:**

The online version of this article (doi:10.1186/s13104-015-1272-z) contains supplementary material, which is available to authorized users.

## Background

Herpes zoster, commonly known as shingles and also known as zona, is a viral disease that is characterized by a painful skin rash with blisters in a limited area on one side of the body, often in the form of a stripe. The initial infection with varicella zoster virus (VZV) causes an acute illness (chickenpox), which generally occurs in children and young people [1].

Of note, VZV can become latent in the nerve cell bodies without causing any symptoms. Years or decades after a VZV infection, however, the virus may cause viral infection of the skin along the corresponding dermatome of the affected nerve [1]. The characteristic symptoms of herpes zoster can make it difficult to distinguish from sciatica induced by spinal diseases, including lumbar disc herniation and spinal canal stenosis [2].

Here we report a case of sciatica mimicking lumbar canal stenosis. The present manuscript confirmed to CARE checklist (Additional file 1).

## Case presentation

A 74-year-old Chinese man sought evaluation at our hospital for left-sided sciatic pain upon standing or walking for 5 min of approximately 1 month’s duration. At the first visit to our hospital, there were no skin lesions. On physical examination, sciatic pain provocation was observed by extension and left bending of the trunk. There was no apparent motor weakness, sensory disturbance, or vesico-rectal disturbance. Bilateral dorsalis pedis artery pulsations were palpable bilaterally. The patient claimed that he was able to ride a bicycle for approximately 1 h.

**Fig. 1:**
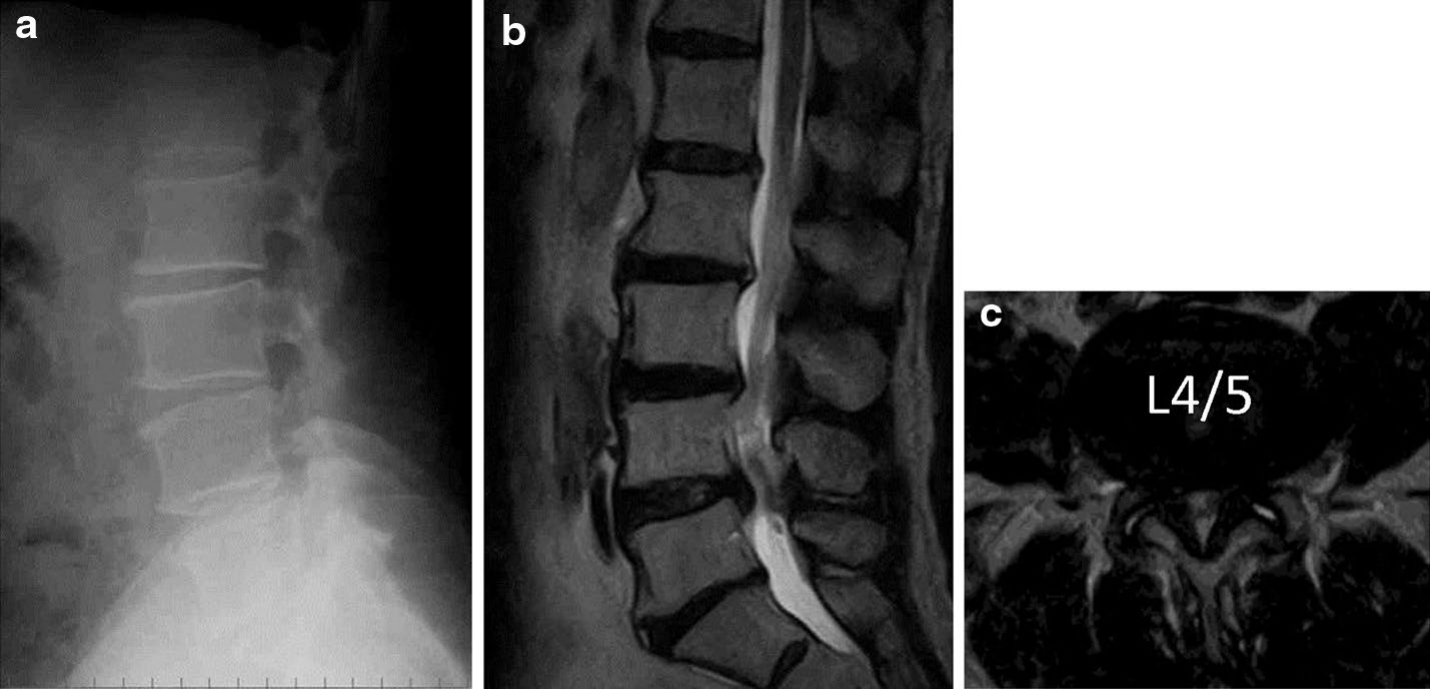
Radiographic findings of the lumbar spine. A plain radiogram of lumbar spine (**a**) showed slight anterior listhesis of the 4th lumbar spine. A magnetic resonance imaging showed spinal canal stenosis between 4th and 5th lumbar spinal level and redundant nerve roots superior to the most stenotic level (**b**, **c**).

A plain X-ray showed slight anterior listhesis of the 4th lumbar (L4) vertebral body (Fig. 1a) without instability on a flexion/extension lateral X-ray. A magnetic resonance imaging (MRI) showed spinal canal stenosis between the 4th and 5th lumbar spine (L4/5) level and redundant nerve roots superior to the most stenotic level (Fig. 1b, c). Thus, we diagnosed the patient with sciatica induced by spinal canal stenosis.

We treated the patient with oral non-steroidal anti-inflammatory drugs (NSAIDS). Because NSAIDS had a minimal effect, a caudal epidural block was attempted using a mixture of a local anesthetic (lidocaine) and steroid. The caudal epidural block provided symptom relief for a few days.

**Fig. 2:**
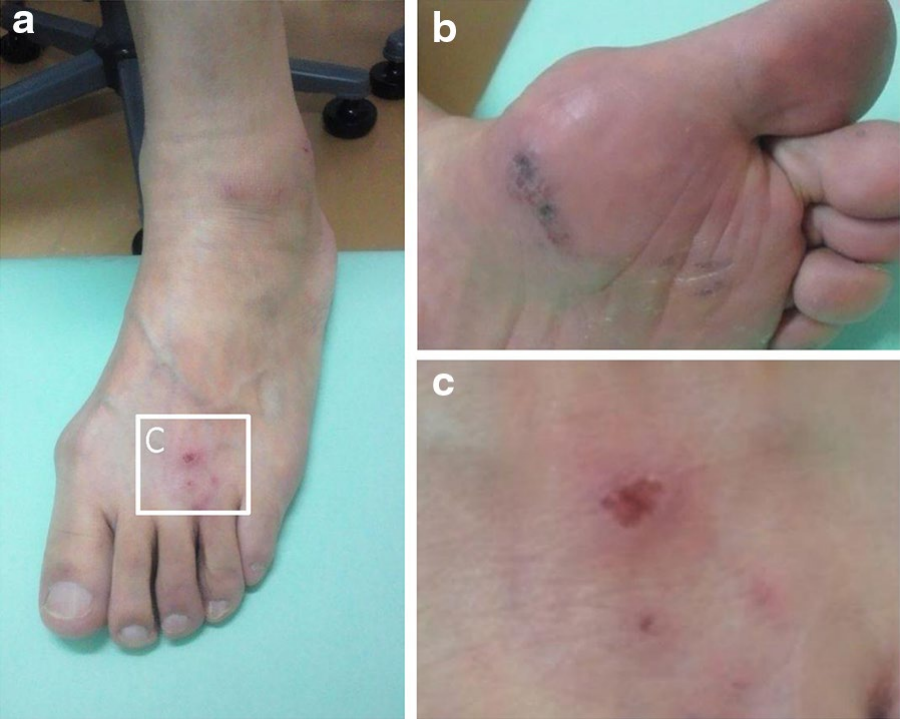
Photograph of skin lesion. A vesicular rash and erythema were observed on the dorsal foot (**a**, **c**) and plantar surfaces of the great toe (**b**) and lateral malleolus (**a**). **c** shows greater magnification of *boxed area* in **a**.

Because repeated caudal epidural blocks (twice in 2 weeks) failed to relieve the symptoms, we considered decompressive surgery for the L4/5 level of stenosis. At that time, the patient complained of a skin rash involving his left foot for several days. A vesicular rash and erythema were observed on the dorsal and plantar surfaces of the great toe and lateral malleolus (Fig. 2). There were no skin rashes involving the buttock, thigh, and lower leg. Pain was diffusely localized to the left foot, lower leg, and lateral thigh. The magnitude of pain was severe, even at rest (85/100 mm on a visual analogue scale of 100 mm). Thus, we consulted a dermatologist in our hospital. The patient was diagnosed with herpes zoster in the left L5 area based on clinical findings, including the characteristics of the pain (along the L5 area) and vesicular rash and erythema in the L5 dermatome.

The patient was treated with famciclovir (1,500 mg/day) and NSAIDs. After 1 week of medication, the skin rash resolved and pain relief was obtained.

## Discussion

The cardinal signs of herpes zoster are neuralgia and skin rash. There is a time lag between the two major symptoms of herpes zoster; neuralgia generally precedes the manifestation of skin lesions for several days to 1 week [2]. The time lag can make it difficult to distinguish sciatica of the lumbar spine origin from herpes zoster infection [2]. There was also a time lag between the neuralgia and skin manifestations in the present case, which led to a delay in diagnosis. In fact, the onset of sciatica preceded the skin lesions by 1 month, which was a protracted time lag compared with previous reports [2]. Therefore, sciatica might be induced by a lumbar spine lesion during the initial period after onset, and might be superseded by a herpes zoster infection during the course of the disease.

There are several reports that have described the differential diagnosis between herpes zoster infection and sciatica of lumbar spine origin. Sprenger De Rover [3] reported that the absence of abnormal MRI findings in the lumbar spine can rule out sciatica of lumbar spine origin. Therefore careful history-taking with respect to the exact nature of the pain and sensory changes is needed to differentiate between herpes zoster infection and radiculopathy if no rash is evident.

If needed, definitive diagnosis of herpes zoster infection can be made by demonstrating viral giant cells using Tzanck’s test, or antibody titers or viral deoxyribonucleic acid levels in cerebrospinal fluid, although negative results do not rule out the diagnosis [1]. In the present case, the characteristic symptoms, including neuralgia and skin rash in the corresponding dermatome, enabled the diagnosis by a dermatologist without further diagnostic tests. The effect of famciclovir supported the diagnosis in the present case.

## Conclusion

In conclusion, spinal surgeons should keep in mind herpes zoster infection as one of the possible differential diagnoses of sciatica, even if there is no typical skin rash.

## Consent

Written informed consent was obtained from the patient for publication of this case report and accompanying images. A copy of the written consent is available for review by the Editor-in-Chief of this journal.

## Additional file

**Additional file 1.** The present manuscript confirmed to CARE checklist.

## Abbreviations

VZV: varicella zoster virus
MRI: magnetic resonance imaging
L: lumbar spine
NSAIDS: non-steroidal anti-inflammatory drugs
DNA: deoxyribonucleic acid
